# COMMUNITY-ENGAGED RECRUITMENT IN PARTNERSHIP WITH FAITH-BASED ORGANIZATIONS: FINDINGS FROM K-HEARS SCREEN

**DOI:** 10.1093/geroni/igae098.0434

**Published:** 2024-12-31

**Authors:** Jami Trumbo, Joshua Betz, Jacqueline Kwak, Joanne Park, Carrie Nieman, Hae-Ra Han

**Affiliations:** Johns Hopkins University, Baltimore, Maryland, United States; Cochlear Center for Hearing & Public Health, Baltimore, Maryland, United States; Johns Hopkins University, Baltimore, Maryland, United States; Johns Hopkins University, Baltimore, Maryland, United States; Johns Hopkins University School of Medicine, Baltimore, Maryland, United States; Johns Hopkins University, Baltimore, Maryland, United States

## Abstract

Hearing loss is highly prevalent, yet few older adults use hearing aids and disparities based on race, ethnicity, and socioeconomic position exist. With limitations in nationally representative data, little is known regarding the hearing health of older Asian Americans, much less among specific ethnic communities. Older Korean Americans (KAs) are predominantly monolingual first-generation immigrants and have some of the highest rates of poverty among Asian Americans as well as high rates of limited English proficiency and poor health literacy, which may contribute to hearing care disparities. The prevalence of hearing loss and hearing aid use among older KAs is not well understood. An observational study was conducted to recruit participants aged 60 and older from KA churches in a metropolitan area to examine the prevalence of hearing loss and hearing care behaviors. A total of 34 recruitment events occurred over 7 months in 16 Korean churches in the Maryland, DC, and Virginia metropolitan area. Screenings occurred in churches of varying congregation size. At least 2 staff members were present at every event (2-4 hour duration). The recruitment efforts resulted in screening 514 KA participants. 13 staff members contributed 414 hours, equating to 0.8 hours of staff effort per individual screened. The KHEARS Screening study represents one of the largest cohorts of KA older adults with hearing loss. Community-engaged research, partnered with faith-based organizations, may offer critical approaches to increasing representation within hearing-related trials and, ultimately, the development of interventions responsive to the needs of a wider population of older adults.

